# Functional pituitary adenoma imaging

**DOI:** 10.1007/s11154-026-10063-4

**Published:** 2026-06-17

**Authors:** Linus Hesse, Linus Haberbosch

**Affiliations:** 1 Charité – Universitätsmedizin Berlin, corporate member of Freie Universität Berlin, Humboldt-Universität Zu Berlin, and Berlin Institute of Health, Department of Endocrinology and Metabolism, European Reference Network On Rare Endocrine Diseases (ENDO-ERN), 10117 Berlin, Germany; 2 Cambridge Endocrine Molecular Imaging Group, Institute of Metabolic Science, University of Cambridge, National Institute for Health Research Cambridge Biomedical Research Centre, Addenbrooke’s Hospital, Hills Road, Cambridge, CB2 0QQ UK; 3 Pituitary Tumor Center of Excellence at Charité – Universitätsmedizin Berlin, corporate member of Freie Universität Berlin, Humboldt-Universität Zu Berlin, and Berlin Institute of Health, Charitéplatz 1, 10117 Berlin, Germany

**Keywords:** Pituitary neuroendocrine tumour, Pituitary adenoma, Cushing’s disease, Acromegaly, Prolactinoma, Positron emission tomography

## Abstract

High-resolution pituitary MRI localises most pituitary neuroendocrine tumours (PitNETs), yet clinically important subgroups remain challenging: (i) very small functioning tumours, particularly corticotroph microadenomas, and (ii) post-operative remnants where scar, gland and tumour cannot be reliably separated. Functional imaging, specifically PET has matured into a problem-solving modality when conventional imaging is equivocal, provided it is used selectively and interpreted within a physiology-led framework. This review proposes an MRI-first, tiered imaging strategy and a pragmatic approach to tracer selection from the available armamentarium ([^11^C]methionine, [^68^ Ga]SSTR ligands, [^68^ Ga]PentixaFor, [^18^F]FET and [^18^F]FDG). We emphasise how to optimise PET acquisition and reporting, and how to integrate imaging with endocrine phenotype, treatment history and surgical/radiosurgical planning to maximise clinical impact while safeguarding pituitary function.

## Introduction

### The persistent clinical gap

Pituitary adenomas or pituitary neuroendocrine tumours (PitNETs) are neoplasms arising from anterior pituitary cells. Although benign in most cases, PitNETs can severely impact patients’ lives through hormone hypersecretion (e.g., Cushing’s disease, acromegaly) or invasive growth leading to visual impairment, cranial nerve damage and hypopituitarism [1]. Neurosurgery is the only curative, and frequently the first-line therapy for most PitNETs [2]. However, without adequate imaging, surgery may be delayed, exploratory, or not feasible at all.

Generally, most PitNETs can be readily visualised on high resolution MRI [1, 3, 4]. However, an important subgroup of patients presents with equivocal or negative MRI findings, and these cases form a challenging, persistent clinical gap in everyday practice of specialist pituitary centres [1, 5–7].

Several scenarios account for this gap: First, radiologically occult or equivocal functioning microadenomas, most notably corticotroph microadenomas, presenting with biochemical-radiological discordance. Indeterminate MRI additionally broadens the differential diagnosis to ectopic ACTH secretion [8, 9]. Second, the post-operative sella, where suspected residual or recurrent tumour needs to be distinguished from scar tissue, packing material, altered gland morphology, and post-surgical enhancement patterns while accurately defining parasellar/cavernous sinus invasion that highly impacts the likelihood of complete resection and subsequent management [10–12].

Inadequate imaging and subsequently indeterminate adenoma localisation necessitates a more extensive neurosurgical sella exploration with an increased risk of collateral pituitary injury (or even calculated (hemi-)hypophysectomy) and subsequent hypopituitarism [13]. Even with more expansive surgery, suboptimal imaging lowers the chances of complete resection and biochemical remission [9, 14]. In cases requiring radiotherapy, indeterminate imaging leads to less focal radiation strategies (larger target volumes and broad-field radiotherapy rather than potentially gland-sparing stereotactic radiosurgery) [13, 15]. Finally, it may lead to reliance on life-long, costly medical therapy, either due to residual disease or potentially avoidable hypopituitarism [16].

### Structural pituitary adenoma imaging

High-quality sellar MRI remains the first-line imaging modality for PitNETs [3]. To ensure optimal results, it should be performed using a dedicated pituitary protocol. This should include high-resolution T1-weighted (T1w) Spin-Echo (SE) MRI in sagittal and coronal planes, before and after gadolinium administration, thin slices (2–3 mm, no interslice gap), high in-plane spatial resolution and a field strength of at least 1.5 Tesla (T); in most centres, 3 T MRI is the standard. Volumetric T1w spoiled gradient-echo sequences (3D-SGE), 3D fast/turbo SE (FSE/TSE) and dynamic contrast-enhanced T1w sequences can improve lesion detection and have been proposed as part of the standard pituitary adenoma work-up [1, 3]. T2-weighted (T2w) sequences can provide valuable complementary information on haemorrhagic, cystic and/or necrotic lesions. Optimal MRI protocols tailored to specific disease entities have been reviewed in detail elsewhere [3, 17, 18]. In certain cases, however, improved MRI sensitivity may come at the cost of reduced specificity. Field strengths of 7 T, where available, can enhance detection of small lesions, but are also more vulnerable to artefacts related to magnetic susceptibility effects, vascular flow and patient motion [3, 4, 6, 7, 17]. Interpretation is further complicated by physiological heterogeneity of normal pituitary gland enhancement [4, 17] and the high prevalence (~ 10% in autopsy studies) of incidental adenomas [1].

Computed tomography (CT) provides inferior soft-tissue contrast compared with MRI, but may still have an adjunctive role when MRI is contraindicated, for the rapid assessment of acute haemorrhage in pituitary apoplexy, and for the detection of calcifications, which are present in up to ~ 80% of craniopharyngiomas [19]. CT may also be indicated to distinguish osseous destruction in aggressive tumours from osseous remodelling in slowly growing lesions. More recently, photon-counting detector CT, with improved spatial resolution and soft-tissue contrast, was reported to achieve a significantly higher detection rate for corticotroph microadenomas than pituitary MRI (92% vs 56% in 25 cases), with fewer misleading (4% vs 24%) and fewer negative scans (4% vs 20%) [20].

Despite these ongoing efforts seeking to reduce the proportion of indeterminate tumours through improved anatomical imaging, an important subgroup of patients remains with indeterminate adenoma localisation. In these circumstances, functional imaging, specifically positron emission tomography (PET), can provide a complementary layer of information [8, 16, 21].

### What functional imaging adds, and what it cannot

When interpreted in conjunction with an accurately co-registered high-resolution MRI, PET can add biological contrast beyond morphological sellar irregularities [21]. The extent of this added value is determined by the tracer’s uptake characteristics in the tissue(s) of interest.

[^11^C]methionine ([^11^C]MET) and [^18^F]fluoroethyl-l-tyrosine ([^18^F]FET) uptake reflects increased amino acid transport consistent with increased peptide synthesis in pituitary adenoma tissue. Visualization of pituitary (and adenoma) tissue can be achieved through direct pituitary receptor imaging with somatostatin receptors (SSTR) targeted [^68^ Ga]DOTATOC, [^68^ Ga]DOTATATE, or [^68^ Ga]DOTANOC or, in corticotropinomas, with [^68^ Ga]DOTA-CRH and [^68^ Ga]Ga-mDesmo. [^68^ Ga]PentixaFor targets the C-X-C motif chemokine ligand 12 (CXCL12)/chemokine receptor 4 (CXCR4) axis, reflecting cell proliferation and possibly also hormone hypersecretion in pituitary and adrenal Cushing’s syndrome. Most broadly used across oncologic indications, [^18^F]FDG provides a marker of increased glucose metabolism as a surrogate of higher metabolic activity in proliferative aggressive pituitary tumours.

With the appropriate tracer selection, patient preparation, and post-image acquisition processing, PET may demonstrate concordant suspicious uptake that increases confidence in a target lesion to cross the threshold for recommending a potentially curative treatment. In selected scenarios with indeterminate conventional imaging findings, the lack of suspicious tracer uptake may also help to distinguish falsely suspected areas such as postoperative change [8, 16, 21].

However, functional imaging also has important constraints. Partial volume effects markedly reduce visual uptake in lesions approaching (or below) the PET system’s effective spatial resolution [22]. Consequently, very small microadenomas (< ~ 6 mm) may be missed despite biological activity and respective tracer uptake [22, 23]. Therefore, absent visual uptake of a single tracer cannot fully exclude low-volume or low-active disease. Additionally, sensitivity is highly dependent on tracer biology, radionuclide physics, and acquisition/reconstruction protocols [23].

A key limitation producing an irreducible component of image blurring is the positron range, as positrons travel a certain distance before annihilating into photons that are detected by the PET scanner. In soft tissue, the median positron range is ~ 2.4 mm for [^68^ Ga] and ~ 0.4 mm for [^18^F] [24], resulting in superior spatial resolution for [^18^F]-labelled tracers [25]. This physical limitation reduces sensitivity for small lesions through partial-volume effects and consequential loss of conspicuity [22]. Conversely, a clearly delineable focal hotspot on a [^68^ Ga]-labelled tracer implies sufficiently high lesion-to-background contrast to overcome blurring; therefore, visually apparent microadenomas on [^68^ Ga] PET constitute a highly specific finding [25]. Overall, the spatial resolution of PET remains inferior to MRI (~ 4 mm vs. ≤ 1 mm) [22, 26].

In most centres PET is acquired as PET/CT and therefore requires accurate co-registration with MRI for anatomical correlation. However, image co-registration is only one component of the technical workflow that can substantially influence the diagnostic value of PET [23]. As most extensively investigated for [^11^C]MET PET in pituitary adenomas, dedicated post-acquisition processing steps—including iterative reconstruction algorithms, normalization to appropriate reference regions, and “delta imaging”—may be important to fully exploit the value of PET imaging.

It is critical to interpret PET findings in the full clinical context. This means not only correlating PET findings to structural imaging, but integrating all available information, including preoperative imaging, operative notes, histology, prior therapies, and importantly, considering the (residual) endocrine activity on the day of the PET scan [26–28].

### Scope and structure of this narrative review

In this review, we aim to present typical opportunities for the use of functional imaging in the diagnostic work-up of pituitary adenomas. We will discuss the use of PET for localising occult microadenomas in Cushing’s disease, as well as discerning residual tumour from postoperative change in residual acromegaly. Alongside these and other cases, we will evaluate the evidence for the principal PET tracers currently used or emerging in pituitary practice—[^11^C]MET, [^18^F]FET, [^68^ Ga]DOTATOC, [^68^ Ga]DOTATATE, [^68^ Ga]DOTANOC, [^68^ Ga]PentixaFor, [^68^ Ga]DOTA-CRH and [^68^ Ga]Ga-mDesmo and [^18^F]FDG with an emphasis on their rationale, strengths and limitations, and clinical questions for which they are most likely to add actionable value. We also summarise practical considerations for optimising case selection, acquisition, processing, interpretation and thereby the impact of functional imaging, embedded within an MRI-first, tiered imaging strategy.

## Cushing’s disease: the occult microadenoma

### The localisation problem in biochemically proven disease

Cushing’s disease is associated with substantial morbidity and excess mortality, yet the corticotroph PitNETs responsible for hypercortisolism are frequently very small microadenomas and MRI-negative in up to 40% of cases [8]. They may be obscured by physiological heterogeneity of pituitary enhancement or overshadowed by incidental sellar findings not causative for hypercortisolism [8, 29]. There is also the differential diagnosis of ectopic ACTH secretion. Current guidelines recommend bilateral inferior petrosus sinus sampling (IPSS) for all suspected corticotroph lesions < 6–9 mm [5]. However, IPSS is invasive, rarely available, and may yield non-diagnostic results. In addition, it is not sufficiently reliable to lateralise tumour within the pituitary gland [5], with correct lateralization rates between 50—70% [30]. This highly limits its ability to guide truly selective surgical resection which is associated with higher cure rates and a lower risk of hypopituitarism [13, 31]. Recently, Lavoilotte and colleagues proposed a urinary free cortisol (UFC)-based strategy for excluding ectopic disease [32]. In their algorithm, an initial UFC level < 10 × the upper limit of normal (ULN) prompts pituitary MRI. If the MRI is positive, no further investigations are recommended, and the patient proceeds to neurosurgery. Otherwise, a neck-to-pelvis CT is recommended. Conversely, a UFC ≥ 10 × ULN leads to CT as first-line imaging, with a pituitary MRI performed only in cases with negative CT findings. If validated in additional cohorts and incorporated into clinical guidelines, this approach could further reduce reliance on IPSS and increase the clinical importance of imaging. While the authors do not mention functional pituitary adenoma imaging in their protocol, the accumulating data and broader availability of a variety of tracers suggests a future role for PET in the diagnostic algorithm for Cushing’s syndrome beyond localising ectopic disease.

### PET tracers for corticotroph tumours

#### Amino-acid tracers: [^11^C]MET and [^18^F]FET

[^11^C]MET and [^18^F]FET PET both exploit increased amino-acid transport via the L-type amino-acid transporter 1 (LAT1) in PitNETs to generate focal uptake in biologically active tumours against lower background activity in the normal pituitary gland. To date, [^11^C]MET is the best-established tracer in functional pituitary imaging [21, 27]. Across most published series, [^11^C]MET PET is positioned as a problem-solving modality for adenoma localisation prior to first surgery in MRI-indeterminate Cushing’s, and as an adjunct in persistent or recurrent disease, where it may support repeat surgical planning by identifying a focal target within a distorted post-operative sella [29, 33–40]. A recent systematic review summarises that 107/124 (86.3%) reported patients with Cushing’s disease had positive results with an added diagnostic value in [^11^C]MET PET [41]. Prospective data emphasize the dependence of [^11^C]MET PET interpretation on MRI and its MR-integrated use for only selected, equivocal MRI patients, with a reported sensitivity of 82% for detecting small corticotroph adenomas [42]. Additionally, PET enabled bypassing invasive IPSS that would have been recommended for all patients in this cohort [5, 42]. Although [^11^C]MET can substantially increase confidence when MRI is equivocal, its pure localisation performance was comparable to the optimised 3 T MRI demonstrating a very high (86%) sensitivity in this cohort—reinforcing the role of PET as a selective adjunct rather than a universal replacement investigation [42]. Dynamic [^11^C]MET PET may further increase confidence in corticotroph tumour localisation, as steeper early uptake slopes and higher peak SUVmean values were observed in tumours compared with the normal pituitary gland [43].

Important pitfalls consist of inadequate anatomical imaging, as CT alone provided insufficient soft-tissue contrast for reliable correlation of PET uptake [44], and the need to assess hormonal status on the day of the scan [45, 46]. The latter is relevant because amino-acid tracer uptake in pituitary adenomas has been hypothesised to be associated with hormonal hypersecretion, suggesting that endocrine activity is necessary for successful tumour localisation [45, 47–49]. This is particularly important in Cushing’s disease, where even very small lesions can cause substantial hormonal excess [35]. However, this relationship remains to be clearly demonstrated. Two recent studies, for example, found no correlation between [^11^C]MET or [^18^F]FET tracer uptake and ACTH or UFC levels in corticotroph tumours [42, 50]. Still, these negative findings also need to be interpreted with caution, because biochemical activity was not assessed on the day of PET in either study. In addition, post-acquisition processing such as PET reconstruction substantially impact the standardised uptake values (SUV) and normalisation to optimal reference structures [cerebellum for [^11^C]MET [47]] was not performed; ongoing medical treatment in ~ 25% of both cohorts may also have introduced heterogeneity.

Future trials need to investigate the potential intraindividual association between [^11^C]MET uptake and endocrine activity, as it would have important implications for improved tumour localisation. The possible utility was recently illustrated by improved tumour detection after initiating treatment with osilodrostat to inhibit negative feedback on a corticotroph adenoma [45]. The lesion was not clearly visualised on the initial [^11^C]MET PET (ACTH: 56.7 ng/l), whereas repeat imaging under osilodrostat with tripled ACTH levels revealed a focal lesion for subsequent transsphenoidal resection [45]. This principle is also utilised in “delta imaging”, in which treatment induced changes in tumour activity compared to a baseline scan may improve lesion conspicuity [47, 48].

Wider adoption and investigation of [^11^C]MET is constrained by its short half-life of only ~ 20 min limiting its availability to centres with an on-site cyclotron. Therefore, [^18^F]FET PET might provide a pragmatic alternative amino-acid tracer if [^11^C]MET is not available. The much longer half-life (~ 110 min) allows its distribution to centres without an on-site cyclotron as well as more flexible scan scheduling. Early clinical data for Cushing’s disease is encouraging, supporting feasibility, clinical impact and good alignment with [^11^C]MET [39, 51–54]. Although tumour uptake may be lower than with [^11^C]MET [55], the typically low physiological background activity of the normal pituitary on [^18^F]FET can preserve lesion conspicuity and thereby help closing the diagnostic gap in previously MRI-occult corticotroph microadenomas. Currently, 50 patients with Cushing’s disease and [^18^F]FET PET have been published across three series [39, 50, 51], and ongoing studies including dedicated prospective evaluations aim to define its possible role in the diagnostic work-up of suspected Cushing’s disease (e.g., NCT07108244 and NCT07360548) and to optimise image acquisition and interpretation. This is especially relevant as the diagnostic value of [^18^F]FET PET is currently limited by physiological, non-specific uptake in the cavernous sinus which can obscure invasive (residual) disease [further discussed below in Sect. 3.3] [56]. If further evidence continues to support its use, the broader availability and also its high resolution as an [^18^F]-labelled tracer may make [^18^F]FET a promising candidate for functional imaging of hypersecretory corticotroph adenomas.

#### CXCR4 targeted PET

[^68^ Ga]PentixaFor is a CXCR4-targeting PET tracer with growing clinical relevance. Initially evaluated in oncology, it is applied in the diagnostic work-up of multiple myeloma, certain lymphomas, and myeloproliferative neoplasms. Outside oncology, it has been shown to improve detection of primary aldosteronism and, notably, adrenal hypercortisolism [57]. It does not require access to an on-site cyclotron, making it a potentially more available tracer. While distribution is currently limited in several regions, its additional diagnostic value in primary aldosteronism could facilitate broader adoption across endocrine centres. Recently, [^68^ Ga]PentixaFor has also emerged as a candidate for detecting corticotroph adenomas [58–60].

While CXCR4 and its ligand CXCL12 contribute to auto- and paracrine regulation within the normal pituitary gland, both have been reported to be overexpressed in pituitary adenomas, with experimental data supporting CXCL12/CXCR4-mediated effects on tumour cell proliferation and hormonal (hyper-)secretion [61].

**Fig. 1:**
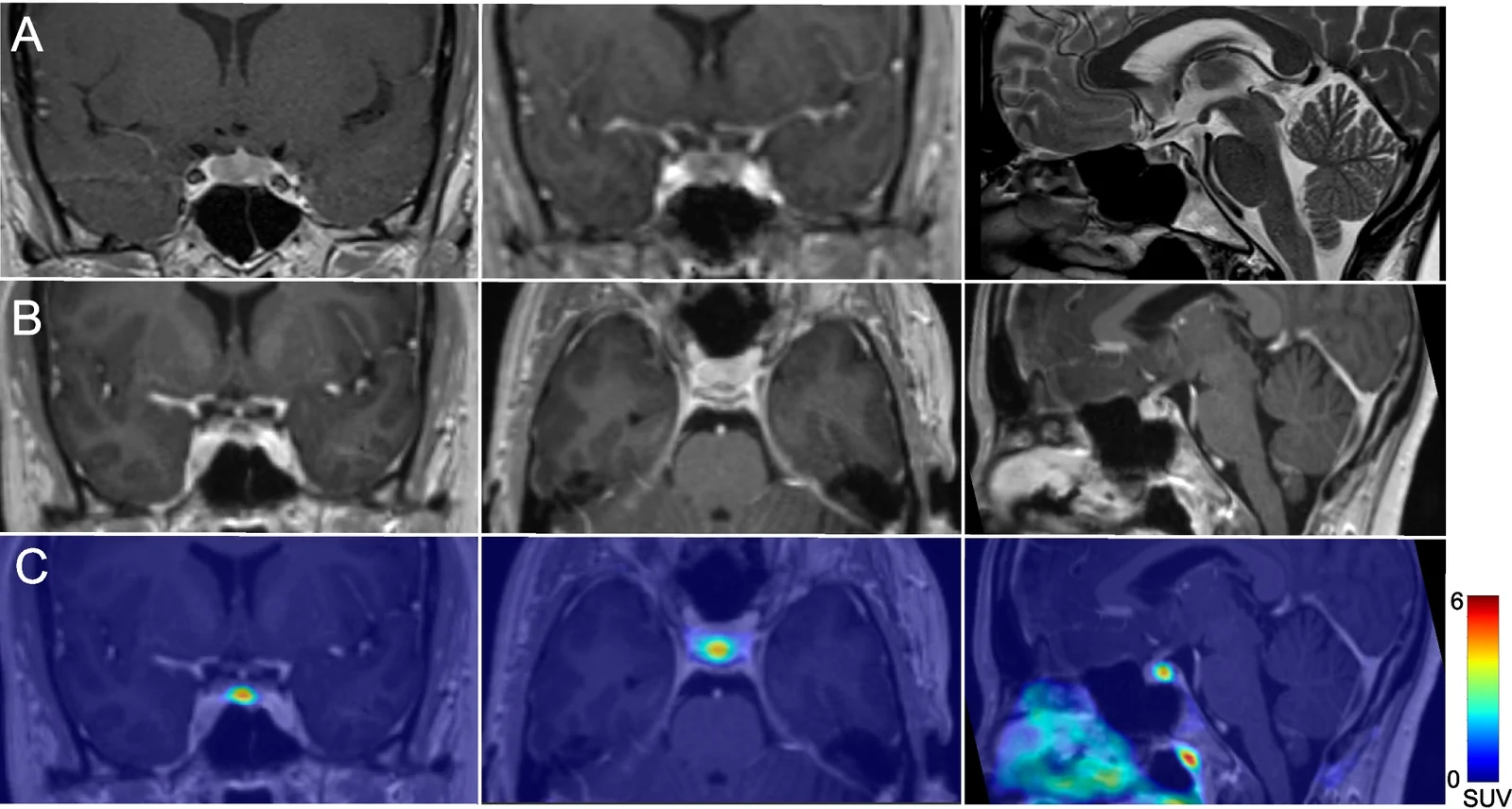
[^68^ Ga]PentixaFor PET/MRI in a patient with cyclical de novo Cushing’s disease. (**A**) Coronal contrast-enhanced T1w TSE and coronal contrast-enhanced dynamic T1w MRI show a small focus of delayed enhancement at the site of stalk insertion. On sagittal T2w TSE, one paramedian hyperintensity and one hypointense focus are seen in the centre of the gland. The sellar findings were previously reported as consistent with a Rathke’s cleft cyst, stable in size over two years. The patient had cyclical Cushing’s disease, which IPSS-confirmed to be central, with otherwise fully preserved pituitary function. Row (**B**), 3D contrast-enhanced T1w gradient-echo MRI demonstrates a small paramedian hypoenhancing lesion within the cleft between the anterior and posterior pituitary lobe. Row (**C**), [^68^ Ga]PentixaFor PET/MRI shows intense focal uptake at the MRI suspicious area, supporting presence of a CXCR4-expressing corticotroph tumour. No tracer uptake is seen in the residual normal pituitary gland as well as very low brain background activity. On the sagittal PET, additional intense tracer uptake is present in lymphoid tissue of Waldeyer’s ring and nasal mucosa, consistent with physiological/inflammatory [^68^ Ga]PentixaFor uptake. Tumour localisation was confirmed intraoperatively, and the patient has shown no evidence of persistent or recurrent ACTH excess at the latest biochemical control (seven months) since surgery. Although definitive histopathological confirmation was not possible, the constellation of preoperative central cortisol excess in IPSS, intraoperative clear tumour visualization at the site of tracer uptake, and postoperative repeatedly shown concordant clinical and biochemical complete remission strongly support the diagnosis of a corticotroph adenoma and its successful removal. Image courtesy of the Department of Nuclear Medicine, Charité – Universitätsmedizin Berlin

In a retrospective series of 78 patients with surgically confirmed corticotroph adenomas, [^68^ Ga]PentixaFor PET/MRI yielded a sensitivity of 98.7%, compared with 75.6% for contrast-enhanced MRI alone [60]. Among a larger cohort of 138 corticotroph adenomas, 43 (31%) exhibited low CXCR4 expression assessed either with low [^68^ Ga]PentixaFor SUVmax or a low h-score on immunohistochemistry. Owing to the very low background CXCR4 uptake reported in the brain and normal pituitary gland [58, 62], a proportion of these lesions may still be visible. Consistently, a second retrospective series, showed no visible pituitary uptake in patients with nonfunctioning pituitary adenomas (n = 4), adrenal adenomas (n = 19 with cortisol producing-, and n = 46 aldosterone producing adenomas) or one patient with ectopic ACTH secretion whereas focal uptake guiding surgical intervention was observed in 86% (6/7) of corticotroph PitNETs [58]. Therefore, beyond pituitary localisation, CXCR4 imaging may also offer diagnostic work-up across the pituitary-adrenal axis in selected scenarios, as high tracer uptake has been described in cortisol producing adenomas of the adrenal glands and at sites of ectopic ACTH secretion [58, 63, 64]. An illustrative case of a corticotroph microadenoma detected on [^68^ Ga]PentixaFor PET is shown in Fig. 1. New [^18^F]-labelled CXCR4 tracers [65] may improve spatial resolution for detecting corticotroph microadenomas. In addition, as proposed in other neuroendocrine tumours [66], positive CXCR4 uptake raises the possibility for CXCR4-directed radionuclide therapy (e.g., with [^177^Lu]/[^90^Y]PentixaTher) in selected, refractory cases [67].

Nevertheless, the early data for CXCR4-targeted PET co-registered with MRI in Cushing’s disease exclusively derive from two Chinese series and therefore require external validation. Moreover, the biological basis for CXCR4-targeted PET regarding its underlying pathophysiologic mechanisms and CXCR4 expression patterns is insufficiently understood. Interestingly, CXCR4 has been predominantly described in somatotroph adenomas, for which no reports of increased [^68^ Ga]PentixaFor PET uptake exist [60, 61]. Clinical adoption will need prospective surgery-confirmed multicentre studies, head-to-head comparisons against the more established tracers [^11^C]MET and [^18^F]FET, and standardised acquisition and interpretation criteria.

#### Direct corticotropinoma receptor imaging: [^68^ Ga]DOTA-CRH and [^68^ Ga]Ga-mDesmo

Two emerging, physiology-linked tracers enable to directly visualise corticotropinoma receptor expression through CRH receptor targeting [^68^ Ga]DOTA-CRH and V1b receptor targeting modified desmopressin with [^68^ Ga]Ga-mDesmo [68, 69].

In a prospective study, [^68^ Ga]DOTA-CRH PET/CT correctly localised 24/24 corticotroph adenomas, including very small lesions (41% < 6 mm) and MRI-negative tumours (17%) despite 3 T volumetric MRI [70]. Importantly, two patients with ectopic ACTH secretion showed no pituitary tracer uptake [70]. Adoption is currently additionally constrained by the limited access to CRH. [^68^ Ga]DOTA-CRH therefore currently remains a promising, but not widely available investigational tracer. Accordingly, published experience is confined to a single centre, which retrospectively reported high background uptake in the pituitary region, potentially limiting lesion-to-gland contrast [71].

[^68^ Ga]Ga-mDesmo PET appears to be a potentially more deployable alternative, with early prospective clinical data reporting delineation in 24/24 patients (33% MRI-negative) [68, 71]. In the same early experience, the absence of pituitary uptake in one inactive pituitary adenoma and in four non-pituitary ACTH-dependent Cushing’s suggests that [^68^ Ga]Ga-mDesmo offers an adjunctive route to selective surgical resection that could reduce reliance on IPSS [71]. Nevertheless, these early encouraging results derive from a single reporting group in a selected subgroup of a rare disease and therefore require external, multicentre confirmation. A further practical limitation is the reported strong bilateral, non-specific cavernous sinus uptake of [^68^ Ga]Ga-mDesmo, which may hinder assessment of parasellar lesions or cavernous sinus invasion and reduce utility in the post-operative, anatomically distorted sella—scenarios in which incremental localisation information is particularly needed.

#### SSTR-targeted PET

Whole-body [^68^ Ga]PentixaFor or whole-body SSTR PET may contribute in cases where ectopic ACTH secretion remains plausible [58, 72]; a systematic review reported localisation of approximately 64% of ectopic ACTH-secreting tumours with SSTR PET/CT [72]. A recent prospective within-patient trial in 24 patients with pathologically confirmed, and cured ectopic ACTH secretion, demonstrated improved ectopic tumour detection with the combination of functional and structural imaging and a sensitivity of 79% for [^68^ Ga]DOTATATE PET/CT alone, not statistically different to an 88% sensitivity for [^18^F]DOPA PET/CT [73], which indicates increased dihydroxyphenylalanine (DOPA) decarboxylase activity across several neuroendocrine tumours [74].

Within pituitary Cushing’s, however, evidence for SSTR PET remains preliminary and context-dependent: Although corticotroph adenomas show lower SSTR tracer uptake than physiological pituitary tissue [28, 75–77], a retrospective study of [^68^ Ga]DOTATOC PET/CT, interpreted blinded to MRI, reported correct corticotropinoma localisation in 77% (26/30) of patients, which was not statistically different than 90% successful localisation of 3 T MRI alone or IPSS with an 68% correct lateralisation rate [28]. Successful localisation appeared to depend more on tumour-to-gland contrast than on absolute tumour uptake, as better diagnostic performance was reported in patients with ACTH > 54 pg/mL, in whom tumour PET uptake was even lower compared to less active adenomas demonstrating more similar uptake compared to the high normal pituitary gland signal [28]. These findings highlight the need for accurate PET/MRI co-registration, given the limited soft-tissue contrast of CT alone and suggest a key limitation of SSTR PET: in an otherwise morphologically normal sella, SSTR PET is unable to reveal MRI-occult corticotroph microadenomas or help de-emphasise indeterminate areas, as the lower tumour uptake will be blunt within high physiological pituitary gland uptake.

#### Metabolic imaging: [^18^F]FDG

Although widely available, [^18^F]FDG PET has not demonstrated superior performance compared with optimised pituitary MRI in the detection of corticotroph PitNETs [5, 78]. Several modulation strategies have been explored: While CRH stimulation might increase detection rate [79], dexamethasone suppression did not result in better corticotroph adenoma localisation [80]. [^18^F]FDG uptake has not been reported to differ significantly between non-functioning adenomas and hormone active adenomas, with a trend to higher uptake in non-functioning adenomas [81, 82]. Accordingly, the most defensible role for [^18^F]FDG PET is not in microadenoma localisation, but rather evaluation of alternative diagnoses—to support confirmation or exclusion of ectopic ACTH secretion and to characterise potentially malignant or inflammatory processes when clinically suspected [83]. In suspected ectopic Cushing’s syndrome, however, positive pulmonary [^18^F]FDG uptake must be interpreted cautiously. A recent review found false-positive uptake in 23% of 106 reported cases, with correspondingly lower specificity for [^18^F]FDG than SSTR PET with only 6% false-positives [84]. Such false-positive findings are mainly attributable to inflammatory pulmonary lesions and may partly be distinguished from true ACTH secreting tumours by their higher SUVmax values [85]. Further pitfalls include physiologically variable [^18^F]FDG uptake in the normal pituitary gland, and non-specific inflammatory uptake in the post-operative sella, which may mimic residual or recurrent tumour [86, 87].

### Integrating PET with endocrine localisation tests and surgical decision-making

In patients with biochemically confirmed ACTH-dependent Cushing’s syndrome, optimised pituitary MRI remains the first localisation step. However, negative or equivocal MRI frequently necessitates additional localisation testing to distinguish pituitary from ectopic ACTH secretion and to inform the surgical approach. CRH and desmopressin stimulation tests, as well as the high-dose dexamethasone suppression test can support this distinction, but discordant results in up to a third of patients and the unavailability of CRH, prompt escalation to invasive IPSS in highly specialised centres [5]. Even in the UFC-based algorithm mentioned above [32], an important subgroup—particularly those with microadenomas presenting with higher UFC values and persistent diagnostic uncertainty [88]—are still suggested to proceed to IPSS [5]. Additionally, patients with a UFC < 3 × the upper limit of normal and negative pituitary MRI as well as neck-to-pelvis CT are proposed to proceed to exploratory neurosurgery.

In these cases, as well as patients who decline IPSS, have contraindications, or in whom IPSS is inconclusive or discordant with the overall clinical picture, PET using [^11^C]MET, [^18^F]FET, or [^68^ Ga]PentixaFor may be considered as a possible adjunctive route to selective surgery. The specific tracer choice will have to include consideration regarding availability ([^11^C]MET), cavernous sinus involvement ([^18^F]FET) and resolution limitations ([^68^ Ga]PentixaFor).

With confirmation from future trials, PET imaging as an adjunct to anatomical imaging might become a key part of future individualized, targeted surgical strategies in (occult) corticotroph tumours [89].

## Acromegaly: the equivocal residual

### Residual disease in altered anatomy

In acromegaly, persistent or recurrent biochemical activity after transsphenoidal surgery is a common and consequential scenario [90]. The diagnostic challenge is frequently not initial detection or the presence of disease, but the precise localisation of active residual tumour within an anatomically altered sella. Post-operative MRI interpretation can be confounded by a mixture of scar tissue, displaced or compressed normal gland, packing material, and post-surgical enhancement, all of which can mimic, obscure, or distort the appearance of small tumour remnants [91]. In addition, subtle parasellar extension—particularly along the medial cavernous sinus wall—may be difficult to distinguish from post-operative change yet is highly relevant for both re-operative feasibility and potentially radiosurgical target definition [91, 92].

Optimised MRI and the selected use of PET enables a higher proportion of patients to not be reliable on long-term medical therapy [12, 93]. Furthermore, imaging can also contribute to therapy selection, for example when considering the likelihood of response to SSTR ligand therapy [94]. However, substantial variability in pituitary imaging protocols within and between centres limits comparability and constrains both routine care and research; accordingly, the recent Acromegaly Consensus Group (ACG) guidance recommends a more harmonised approach to pituitary imaging [90].

### [^11^C]MET PET: best available evidence

Regarding functional imaging, the ACG guidelines specifically mention [^11^C]MET PET, which is deemed helpful for localising residual disease as well as rare occult adenomas [90]. Indeed, [^11^C]MET PET currently has the strongest clinical evidence base among PET tracers for acromegaly and is most consistently positioned as a problem-solving adjunct when MRI is indeterminate, but identification of a focal target would change management. Across > 100 reported acromegalic cases, [^11^C]MET typically demonstrates focal uptake indicating viable somatotroph tumour tissue [12, 35, 38, 93, 95]. In a cohort in which > 90% of patients had indeterminate or negative MRI, [^11^C]MET PET-guided repeat surgery achieved complete biochemical remission in 24/33 (73%); the remaining patients showed significant biochemical improvement, with no apparent excess risk of hypopituitarism after repeat surgery [12]. These outcomes notably exceed benchmarks: a meta-analysis reported a mean remission rate of 44.5% after second transsphenoidal surgery for growth hormone secreting tumours in cohorts predominantly comprising MRI-visible residual adenomas [96]. Although [^11^C]MET PET identified a potential target in 85% of the overall cohort, only 54% of patients underwent PET-guided surgery. This highlights that [^11^C]MET PET findings should be integrated with all available clinical, biochemical, and imaging information within MDT-based selection for reoperation, including tumour invasiveness and neurosurgical expertise. While these additional selection factors limit the ability to establish a definitive causal relationship of PET leading to improved outcomes in MRI-equivocal cases, PET findings enabled definitive treatment in patients who might otherwise not have been considered surgical candidates [12].

[^11^C]MET PET is ideally performed once post-operative anatomy has stabilised as it depends on high-quality interpretable MRI for accurate anatomical correlation and when there is concurrent biochemical evidence of persistent disease. Accordingly, imaging within very early post-operative windows (< 3 months) is usually avoided in most cases where non-specific changes in MRI and PET may confound interpretation, or initial remission is falsely suspected because only microscopic adenoma cells, below the threshold for PET detection, were not resected [22, 97]. Medical therapy can suppress tumour activity and potentially reduce tracer conspicuity; therefore, pragmatic drug washout strategies are recommended, for example discontinuation of depot somatostatin analogue therapy for at least 12 weeks (and dopamine agonists for ~ 4 weeks) prior to [^11^C]MET PET, to maximise the likelihood of detecting residual tumour [12].

### [^18^F]FET PET where [^11^C]MET is unavailable

Analogous to the proposed approach in Cushing’s disease, [^18^F]FET PET can be positioned as a pragmatic alternative amino-acid tracer within a similar problem-solving framework in MRI indeterminate residual or recurrent acromegaly, if a focal target would meaningfully change management (repeat surgery vs. stereotactic radiosurgery vs. escalation of medical therapy). The evidence base is substantially smaller than for [^11^C]MET PET; emerging data suggest that [^18^F]FET PET co-registered with high-resolution MRI can positively influence decision-making in patients with persisting acromegaly and no clear surgical target on MRI [98–100].

A major interpretive pitfall of [^18^F]FET PET is its physiological, non-specific tracer uptake in the cavernous sinus, which can confound assessment of parasellar disease and complicate lateralisation [56]. This limitation is particularly relevant in somatotroph PitNETs, which frequently invade the cavernous sinus; with reported rates of cavernous sinus invasion reaching up to ~ 56% at diagnosis [101]. In the post-operative sella—where anatomy is distorted and residual tumour often abuts the cavernous sinus—this ambiguity can be decisive, because the feasibility of complete resection at re-operation depends on accurate delineation of parasellar disease and may drive the choice between repeat surgery, radiosurgery/radiotherapy, and long-term medical therapy [11]. The mechanisms of non-specific [^18^F]FET accumulation in the cavernous sinus are not yet fully elucidated. Unlike [^11^C]Met, [^18^F]FET is not incorporated into peptides. Consequently, Bakker et al. hypothesized that unmetabolized [^18^F]FET may therefore wash out through the reversible L-type amino acid transport system and pool in the cavernous sinus [98]. Reference data, however, is lacking, and pituitary-optimised acquisition protocols remain important to be defined with the increasing use of [^18^F]FET in Cushing’s and acromegaly [98, 102].

### [^68^ Ga]SSTR PET: receptor mapping and selected localisation questions

SSTR PET using [^68^ Ga]-labelled SSTR binding tracers ([^68^ Ga]DOTATATE, [^68^ Ga]DOTATOC and [^68^ Ga]DOTANOC) can offer complementary information to MRI. Its most defensible role is therapeutic planning in complex or refractory disease, where SSTR mapping may inform multidisciplinary decision-making [103, 104] and, in selected aggressive adenomas or pituitary carcinomas, may contribute to theranostic considerations regarding peptide receptor radionuclide therapy (PRRT) eligibility with [^177^Lu]DOTATATE or [^90^Y]DOTATATE [105, 106]. Importantly, current evidence does not support using SSTR PET to predict the response to somatostatin receptor ligand (SRL) therapy [107, 108]. This is consistent with earlier SSTR scintigraphy studies, which also failed to show consistent predictive value [109, 110]. Nevertheless, [^68^ Ga]DOTATOC SUVmax was shown to correlate strongly with SSTR2 immunoreactivity (r = 0.75) in pituitary adenomas, whereas no correlation was observed for all other SSTR subtypes [75]. In a small acromegaly series using [^68^ Ga]DOTATATE with an even ~ tenfold higher SSTR2 affinity than [^68^ Ga]DOTATOC, only a non-significant trend towards higher SUVmax in complete SRL responders (p = 0.0576) was shown, although SUVmax correlated inversely with post-treatment GH levels (r = − 0.60) [107]. In parallel, the randomised, placebo-controlled GALANT trial, including only SSTR expressing *non-functioning* pituitary macroadenomas confirmed by [^68^ Ga]DOTATATE uptake with adenoma SUVmean > 2, showed no tumour growth benefit after 72 weeks of lanreotide versus placebo [108].

Moreover, the utility of SSTR PET for adenoma localisation is constrained by physiology and tumour subtype, and data regarding adenoma uptake as compared to the high physiological pituitary gland uptake is based on only small case series [75, 111]. The high physiological SSTR ligand uptake in normal pituitary gland tissue can obscure adenoma visualization, particularly in microadenomas of an untreated sella [76]. Moreover, somatotroph adenomas frequently demonstrate high SSTR tracer uptake that may not differ sufficiently from normal pituitary uptake to enable confident lesion delineation, whereas clearer contrasts have been reported for other adenoma subtypes (e.g., higher uptake in thyrotroph tumours, and significantly lower uptake in corticotroph and non-functioning adenomas) [28, 75, 111]. Furthermore, because activated inflammatory cells (particularly macrophages) express SSTR2, SSTR-targeted tracers can show non-tumour uptake in inflammatory lesions, which can be a potential source of false-positive signal [112]. Accordingly, SSTR PET should only be recommended selectively—most appropriately when it clarifies a pre-defined localisation question that is likely to change management [26].

A practical advantage of SSTR tracers is their very low non-specific brain uptake compared with amino-acid tracers and [^18^F]FDG [113, 114]. Consequently, SSTR PET is best reserved to clarify specific questions, most commonly in pre-treated patients with complex sellar anatomy: (i) it may increase confidence in defining parasellar extension, including suspected cavernous sinus involvement, thereby informing feasibility of repeat surgery, (ii) it can support radiosurgical target definition by helping to distinguish active tumour from postoperative change and (iii) can identify the normal pituitary remnant, e.g. to facilitate gland-sparing irradiation planning and reduce the risk of post-radiation hypopituitarism [56, 115].

### PET in acromegaly management

**Fig. 2:**
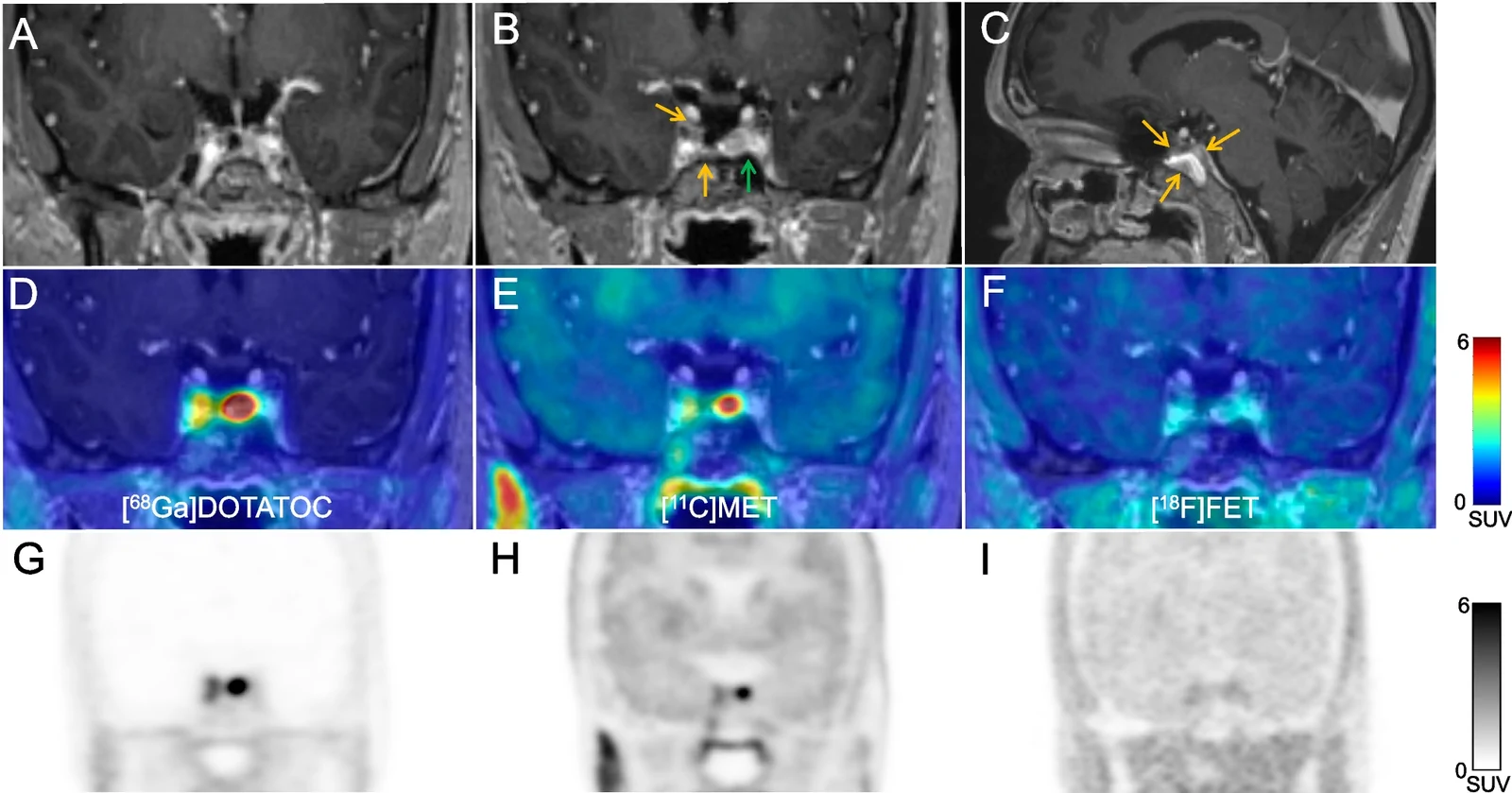
[^68^ Ga]DOTATOC, [^11^C]MET and [^18^F]FET PET/MRI in a patient with residual acromegaly. In a patient with residual acromegaly following two transsphenoidal surgeries with tumour resection extending to the medial walls of both cavernous sinuses, anterior pituitary function remained fully preserved. Coronal and sagittal contrast-enhanced T1w 3D gradient-echo MRI (**A**–**C**) demonstrates residual tumour invading the right cavernous sinus (yellow arrows in **B** and **C**), and likely residual normal pituitary gland in the left sella. Co-registered PET/MRI with [^68^ Ga]DOTATOC (**D**), [^11^C]MET (**E**) and [^18^F]FET (**F**) illustrates tracer-specific patterns: [^68^ Ga]DOTATOC PET (**G**) and [^11^C]MET PET (**H**) show high physiological uptake in the residual normal pituitary gland, consistent with the fully preserved pituitary function, whereas [^18^F]FET PET (**I**) shows no normal gland uptake. The [^68^ Ga]DOTATOC hotspot (**D**, **G**) appears larger than the [^11^C]MET focus (E, H), consistent with the lower spatial resolution of [^68^ Ga]-labelled tracers. [^68^ Ga]DOTATOC and [^11^C]MET show focal uptake in the right cavernous sinus concordant with the MRI-suspicious areas, albeit lower than physiological uptake in the normal gland. [^18^F]FET shows only diffuse low-grade activity in both cavernous sinuses (**F**, **I**). This case has been previously reported in the Journal of Nuclear Medicine [56]. The current figure presents additional images not included in the earlier publication

In conclusion, PET is most useful in residual or recurrent disease, where the central challenge is not confirming persistence but localising viable tumour within a complex post-operative sella. [^11^C]Met is showing the best evidence in acromegaly, although each tracer has distinct strengths and limitations. Figure 2 shows a case of a patient with residual acromegaly undergoing [^11^C]Met PET as well as [^18^F]FET PET and [^68^ Ga]DOTATOC PET, highlighting the different advantages and challenges of these tracers in diagnosing somatotroph tumours. While prospective trials are needed, PET can improve localisation confidence for (residual) somatotroph tumours, potentially leading to focused re-operation or precise radiosurgical contouring and thereby curative treatment.

## Prolactinoma: neurosurgery ascending

### Why imaging precision is becoming more clinically relevant

Historically, prolactinomas have been managed predominantly with dopamine agonists, and imaging has largely served a confirmatory and surveillance role rather than acting as the primary determinant of definitive therapy. Surgical intervention was mostly confined to more aggressive prolactinomas with optic pathway affection, which are readily visualised on MRI. However, the recent Pituitary Society consensus guidance supports considering transsphenoidal surgery as a first-line treatment in selected prolactinoma patients [116], increasing the importance of precise anatomical target definition. For small intrasellar tumours, experienced neurosurgeons can achieve high remission rates with low morbidity, offering a definitive alternative to life-long medical therapy [116, 117]. Surgery is also an important option when dopamine agonists are ineffective or poorly tolerated by neuropsychiatric adverse effects. The decision for a neurosurgical intervention, however, requires careful consideration of potential success weighted against possible complications. Postoperative hypopituitarism will just result in the patient switching one life-long medication for another and might not improve their quality of life at all. Accordingly, careful delineation of lesion boundaries, their relationship to the cavernous sinus and (residual) normal pituitary gland, and confirmation of a discrete, surgically accessible target, provide a rationale for an MRI-first but escalation-ready imaging strategy in the current care of prolactinoma patients.

### PET approaches in prolactinoma pathways

#### Amino-acid tracers: [^11^C]MET and [^18^F]FET

Prolactinomas typically show high uptake on [^11^C]MET PET, as highlighted by three prolactinoma [^11^C]MET PET series published to date, which report clinical utility/positive localisation in 94% (17/18) [118], 100% (13/13) [119], and 78% (7/9) [38] of patients with dopamine agonist intolerance/resistance and equivocal MRI. The absolute number of published PET-guided, surgically cured prolactinoma cases remains small, reflecting the predominantly medical treatment but also the more restrictive study inclusion criteria of tiered PET imaging reserved for patients with equivocal MRI *combined with* dopamine agonist intolerance/resistance.

Being aware of its limitations with lower adenoma uptake and physiological cavernous sinus activity—with current acquisition protocols prohibiting potential exclusion of tumour tissue in the cavernous sinus—[^18^F]FET PET appears to offer a logistically more accessible alternative within a similar MRI-first, tiered strategy when [^11^C]MET PET is not feasible [118]. Evidence is currently restricted to 20 patients from two series [50, 120], but early results are encouraging: the principal incremental value was the identification of new surgical targets in 88% (15/17), with surgical success in 7/8 (88%) operated patients [120]. As in other functioning PitNETs, optimising pre-test conditions is important; where clinically safe and feasible, temporary discontinuation of dopamine agonists 4 weeks prior to amino-acid PET is recommended to minimise tumour suppression and maximise detectability. Interpretation should also be embedded within a systematic re-evaluation of all available information—including longitudinal MRI from diagnosis to the most recent scan, biochemical activity, prior operative reports, and histology/immunohistochemistry—to increase the probability of PET contributing actionable incremental value.

#### Dopamine receptor imaging

Targeting the dopamine D2 receptor is biologically attractive in prolactinomas because it directly interrogates the molecular target of the first-line medical therapy. D2-targeted PET tracers such as [^11^C]raclopride and [^18^F]fallypride have been explored in small studies, and showed potential predictive value for dopamine agonist response in prolactinomas (and non-functioning adenomas) [27, 121–123]. However, dopamine receptor PET remains highly selective: wider adoption is constrained by limited availability and absence of standards for acquisition and interpretation [27]. Moreover, the currently existing, heterogeneous case studies have not demonstrated that dopamine receptor imaging improves clinical outcomes beyond what can typically be achieved through endocrine phenotype, treatment history, high-quality MRI or, if necessary, escalation to amino acid PET [49, 122, 124]. Accordingly, dopamine PET is reserved for niche scenarios, such as unclear dopamine agonist resistance or intolerance where receptor characterisation could plausibly influence escalation towards surgery, or alternative medical strategies within specialist multidisciplinary care pathways.

## TSH-oma and other rarities

### TSH-oma: why functional imaging can be particularly helpful

Although the rarity of TSH-omas inevitably limits cohort sizes, effect sizes can still be clinically meaningful when PET is applied selectively to MRI-equivocal cases. [^11^C]MET has repeatedly demonstrated high tumour-to-gland contrast in case reports and small series, revealing discrete microadenomas previously not identified confidently enough on conventional MRI to enable targeted surgery [47, 125]. In a case series, the [^11^C]MET-suspicious site was histologically confirmed with thyrotroph tumour in all (8/8) surgically treated patients [47]. In addition, SSTR PET can be informative in thyrotroph adenomas. Compared with corticotroph and non-functioning PitNETs—where SSTR ligand uptake is lower compared to the residual normal pituitary—thyrotroph tumours have been reported to show very high uptake of SSTR ligands exceeding normal pituitary background and thereby facilitating delineation of tumour tissue [75, 103, 126].

### Other rare but high-impact scenarios

In the setting of metastatic disease, management and diagnostic pathways rely on functional imaging, but they differ fundamentally from pituitary adenomas with respect to systemic staging and multimodal oncologic treatment; a detailed discussion is therefore beyond the scope of this review.

For aggressive pituitary tumours, defined by rapid tumour progression or clinically relevant progression despite optimal therapy, the European clinical guideline recommends whole body screening, e.g., with [^18^F]FDG or SSTR PET in all patients with either biochemical-radiological discordance, site-specific symptoms, or before chemotherapy [127]. In this context, receptor status is highly heterogeneous, nonetheless SSTR PET, and for aggressive corticotroph tumours possibly also [^68^ Ga]PentixaFor PET, aid both lesion detection within whole-body staging and the identification of candidates for theranostic approaches such as PRRT [58, 67, 105, 127].

When [^18^F]FDG PET is performed for staging of non-pituitary malignancies, the pituitary should be systematically reviewed, particularly if immune checkpoint inhibitor (ICI) therapy has been applied or might be a future option. Pituitary [^18^F]FDG uptake has been reported to rise in the weeks preceding clinically apparent ICI-related hypophysitis, which occurs in up to ~ 10% of ICI-treated patients [81, 128–130]. Although potentially life-threatening through central adrenal insufficiency, ICI-related hypophysitis is well treatable with appropriate hormonal replacement and, importantly, is not considered a general contraindication to ICI continuation [131]. Future studies should investigate a possible role of PET in predicting ICI-related hypophysitis and potentially identifying patients at risk of suffering an adrenal crisis after therapy.

## When and how to use PET for maximum effect

### Optimising tracer selection, PET acquisition, interpretation and reporting

An overview of best-fit scenarios for PET tracer selection in PitNETs is provided in Table 1*.* Patient preparation should be tracer-specific and aligned with the pre-specified clinical question. Standardised reporting necessitates a basal pituitary biochemistry on the day of PET. Potentially confounding, adenoma-directed medication should be reviewed and, where clinically safe, a discontinuation of depot somatostatin analogue therapy for at least 12 weeks, and dopamine agonists for ~ 4 weeks prior to amino-acid PET, has been used to increase the likelihood of detecting active tumour tissue [12]. Treatments altering endocrine drive, such as metyrapone/osilodrostat in Cushing’s and pegvisomant in acromegaly, may also increase tumour and normal gland uptake [45, 46]. Given the complexity and the potential for false attribution, PET interpretation is best performed within a dedicated pituitary MDT, integrating nuclear medicine, neuroradiology, endocrinology, neurosurgery and radiosurgery expertise.

**Table 1:** Overview of the currently emerging tracers in pituitary practice

Tracer	Target	Proposed Indications	Advantages	Challenges	Evidence	Availability
[^11^C]MET	L-type amino acid transporter 1 (LAT-1), incorporated into peptides	➢ lesion localisation and delineation, including small functioning adenomas prior first surgery and post-surgery ⊕⊕⊕ O ➢ extent of cavernous sinus invasion ⊕⊕⊕ O ➢ confirming remission with a baseline scan or with targeted assessment of a suspected residual lesion ⊕OOO ➢ detecting recurrence ⊕⊕⊕ O ➢ can reduce the need for IPSS ⊕OOO	➢ uptake directly consistent with hormonal hypersecretion and endocrine activity ➢ better spatial resolution than [^68^ Ga] ➢ optimised through normalization, e.g., enabling delta imaging ➢ lower radiation exposure compared to tracers with longer half-life	➢ unflexible scan scheduling with 20 min half-life ➢ normal gland uptake higher than [^18^F]FET	➢ the best studied tracer in Cushing’s, Acromegaly, Prolactinomas and TSHomas ➢ retrospective, surgically confirmed cohort studies and case series with n > 274 reported cases [41] ➢ one prospective multicentre evaluation (n = 30)	➢ highly limited, requires on-site cyclotron
[^18^F]FET	L-type amino acid transporter 1 (LAT-1), not incorporated into peptides	➢ Localisation and lesion delineation of small functioning tumours prior first surgery and post-surgery when [^11^C]MET is unavailable ⊕⊕ OO ➢ can reduce the need for IPSS ⊕OOO	➢ low physiological pituitary gland uptake ➢ highest spatial resolution with [^18^F] ➢ half-life ~ 110 min allows more flexible scan scheduling	➢ lower tumour uptake compared to [^11^C]MET ➢ Non-specific, physiological cavernous sinus uptake	➢ limited and exclusively retrospective surgically confirmed cohorts with n < ~ 100 reported cases and case reports	➢ more available than [^11^C]MET, more broadly used in brain tumours ➢ no on-site cyclotron required
[^68^ Ga]DOTATOC, [^68^ Ga]DOTATATE, [^68^ Ga]DOTANOC	Somatostatin receptor (SSTR)	➢ selected localisation questions prior first surgery: suspected macro-lesion not within the normal gland, post-surgical cavernous sinus invasion, differential diagnosis to postoperative change ⊕OOO ➢ radiosurgical planning in the dispersed sella ⊕OOO ➢ staging of aggressive tumours ⊕⊕⊕ O	➢ very low brain background uptake for the delineation of parasellar disease and cavernous sinus invasion ➢ very high uptake in TSHomas ➢ theranostic potential	➢ high normal gland uptake ➢ low tumour uptake (except TSHomas) ➢ not a universal microadenoma localisation tool ➢ inflammatory uptake ➢ Low [^68^ Ga] resolution	➢ limited data for single tumour entities ➢ mainly retrospective cohorts with < ~ 200 reported cases	➢ generator-produced; no cyclotron needed broadly available in NET centres
[^68^ Ga]PentixaFor	C-X-C motif chemokine receptor 4 (CXCR4)	➢ corticotroph lesion localisation ⊕⊕ OO ➢ can reduce the need for IPSS ⊕OOO	➢ assesses endocrine differential diagnoses ➢ low gland background uptake ➢ no uptake in other adenoma entities including nfpa ➢ theranostic potential	➢ only for Cushing’s ➢ low [^68^ Ga] resolution	➢ limited to two Chinese series with n = 85 scanned corticotroph adenomas in total	➢ generator-produced only in specialised centres; variable access with oncologic and endocrine indications
[^18^F]FDG	glucose transporters (GLUTs)	➢ aggressive tumours ⊕⊕ OO ➢ malignancy, staging, metastatic work-up ⊕⊕ ⊕ O ➢ ectopic search in selected cases ⊕⊕OO ➢ inflammation/ICI induced hypophysitis ⊕OOO	➢ highest spatial resolution with [^18^F] ➢ most flexible scan scheduling	➢ unspecific, physiologically heterogeneous uptake patterns ➢ no routine localisation tool ➢ fasting ≥ 6 h and adequate glycaemic control required	➢ highly problem-specific evidence base: ➢ metastases/cancer > aggressive adenomas > ICI induced hypophysitis > pituitary adenomas	➢ Best availability, broadest indications

Each proposed indication includes an estimate of its corresponding evidence level, using the following grading system: very low (⊕ OOO), supported by case reports or a single small retrospective series; moderate (⊕ ⊕ OO), supported by several smaller retrospective cohort studies; and high (⊕ ⊕ ⊕ O), supported by prospective cohort studies or by several larger retrospective cohorts

PET/CT requires accurate co-registration to a dedicated thin-slice volumetric pituitary MRI; alternatively, PET/MRI can be performed. Partial-volume effects limit detection of very small lesions (≤ ~ 6 mm) with low uptake [22]. However, for [^11^C]MET—the best studied and optimised tracer in PitNETs—increased accuracy including more sensitive visualization of small adenomas was demonstrated through a study with bespoke 3D printed radioactive skull phantoms with different sizes of pituitary adenomas: Bayesian penalized likelihood iterative reconstruction algorithms with time of flight, point spread function correction and further optimised PET scanner parameters all demonstrated increased sensitivity [23]. Additionally, normalization to reference regions can reduce inter-scan and inter-individual variability and enable standardised, more reproducible interpretation [47]. For [^11^C]MET, normalisation to cerebellum cortex has been shown to mitigate the physiological variation in pituitary uptake (≥ 20% of SUVbw in healthy pituitary glands) and to facilitate intra-individual “delta” comparisons (e.g., on versus off SRLs or osilodrostat) that can enhance detection in borderline cases [45, 47, 48]. For SSTR PET, normalization to the superior sagittal sinus, as a reference of the cranial blood pool has been reported to reduce uptake variability [132]. Thereby, normalization may also enable standardised and reproducible windowing, a variable that remarkably influences visually assessed adenoma volume and delineation in irradiation planning [133]. This might be particularly helpful for irradiation planning in the post-operative dispersed sella that shows equivocal radiosurgical targets on MRI alone [56, 115]. In selected cases, complementary PET with different tracers may be justified to separate tumour signal and localise normal gland tissue (see Fig. 2) [40, 104, 134].

Overall, PET should be positioned as an adjunct—not a replacement—for comprehensive clinical evaluation, including optimised, high-resolution pituitary MRI and rigorous endocrine phenotyping. Its added value is greatest when it is used to resolve a specific, pre-defined uncertainty and is interpreted within the complete biochemical work-up.

Future progress in PET imaging for PitNETs will depend on standardisation of acquisition, co-registration, interpretation and reporting, enabling meaningful multicentre comparisons and evidence synthesis [26]. Technological advances, particularly the increasing availability of total-body PET scanners, are expected to demonstrate improved sensitivity. Prospective, surgery-confirmed cohorts and head-to-head tracer comparisons are warranted for further evidence-based application of PET in MR-indeterminate PitNETs.

## Conclusion

Functional imaging is rapidly becoming a relevant part in the diagnostic workup of challenging pituitary adenomas. While the occult corticotroph adenoma is the most obvious application, certain cases of residual somatotroph tumours and other pituitary adenomas can also profit from PET imaging identifying a surgical target and enabling potentially curative neurosurgery. The future will likely not be a single winning tracer for all pituitary adenomas, but an individualised strategy: MRI-first, tiered escalation, and physiology-led as well as evidence-based tracer choice, optimising and integrating all available information to guide the most definitive, pituitary-sparing intervention.

## Data Availability

No datasets were generated or analysed during the current study.

## Abbreviations

[^11^C]MET: [^11^C]methionine
[^18^F]DOPA: [^18^F]fluorodopa
[^18^F]FDG: [^18^F]fluorodeoxyglucose
[^18^F]FET: [^18^F]fluoroethyl-L-tyrosine
ACG: Acromegaly Consensus Group
ACTH: Adrenocorticotropic hormone
CRH: Corticotropin-releasing hormone
CT: Computed tomography
CXCL12: C-X-C motif chemokine ligand 12
CXCR4: C-X-C motif chemokine receptor 4
DOPA: L-3,4-dihydroxyphenylalanine
IPSS: Inferior petrosal sinus sampling
MRI: Magnetic resonance imaging
PET: Positron emission tomography
PitNET: Pituitary neuroendocrine tumour
SE: Spin echo
SRL: Somatostatin receptor ligands
SSTR: Somatostatin receptors
T1w: T1-weighted
T2w: T2-weighted
